# Increased expression levels of the *pvcrt-o *and *pvmdr1 *genes in a patient with severe *Plasmodium vivax *malaria

**DOI:** 10.1186/1475-2875-8-55

**Published:** 2009-04-02

**Authors:** Carmen Fernández-Becerra, Maria Jesús Pinazo, Ana González, Pedro L Alonso, Hernando A del Portillo, Joaquim Gascón

**Affiliations:** 1 Barcelona Centre for International Health Research (CRESIB), Hospital Clinic/IDIBAPS, Universitat de Barcelona, Roselló 132, 4a planta, 08036, Barcelona, Spain; 2 Catalan Institution for Research and Advanced Studies (ICREA), Passeig Lluís Companys, 23 08010 Barcelona, Spain

## Abstract

**Background:**

There are increasing reports of severe clinical cases exclusively associated with *Plasmodium vivax *infections. Notably, this severity has been recently suggested to be associated with chloroquine resistance.

**Patients:**

Two different patients presented at the Hospital Clinic in Barcelona with *P. vivax *malaria episodes. One patient had severe symptoms and the other mild symptoms. Both patients traveled through the Brazilian Amazon (Manaus) in 2007. For both patients the current diagnosis of malaria was the first. Two other patients with mild symptoms presented to the "Centro de Pesquisa em Medicina Tropical", also in the Brazilian Amazon (Rondônia) in 2000.

**Methods:**

To exclude the possibility that the patient's severe symptoms were due to *Plasmodium falciparum*, a nested PCR was performed. A magnetic method was used to purify *P. vivax *free of human leukocytes. Quantitative real-time PCR was performed to compare the transcript levels of two main transporters likely to be involved in chloroquine resistance in *P. vivax*, namely the *P. vivax *chloroquine resistance transporter, *pvcrt-o*, and the *P. vivax *multidrug resistance transporter, *pvmdr 1*.

**Results:**

Results demonstrated that the severe clinical symptoms were exclusively due to *P. vivax*. The patient presented acute respiratory conditions requiring admission to the intensive care unit. The magnetic method showed highly purified infected-reticulocytes with mature stages. In addition, it was found that parasites obtained from the severe patient had up to 2.9-fold increase in *pvmdr1 *levels and up to 21.9-fold increase in *pvcrt-o *levels compared to expression levels of parasites from the other patients with mild symptoms.

**Conclusion:**

This is the first clinical case of severe disease exclusively associated with vivax malaria in Spain. Moreover, these findings suggest that clinical severity could be associated with increased expression levels of parasite genes likely involved in chloroquine resistance. It is necessary to further explore the potential of *pvmdr1 *and particularly *pvcrt-o *expression levels as molecular markers of severe disease in *P. vivax*.

## Background

The renewed momentum for global malaria eradication has highlighted the need to further studies on *Plasmodium vivax *if eradication is to be achieved. Although the exact burden of disease is still a matter of debate, it is likely that it has been underestimated and that *P. vivax *is responsible for between 100 and 300 million clinical infections each year [1]. The emergence of worsening clinical severity and chloroquine resistance are two major factors responsible for this increasing burden.

*Plasmodium vivax *infections have been associated with mild symptoms such as fever, headache, fatigue, chills, and musculoskeletal pain, in particular paroxysms. Recently, however, severe complications, including renal failure, jaundice, acute respiratory distress syndrome, cerebral malaria, seizures, anaemia, hyperparasitaemia, thrombocytopenia, pulmonary edema, splenic rupture and death, have been reported in exclusive association with *P. vivax *[2,3].

Chloroquine resistance (CQR) is a major determinant of the present resurgence of malaria worldwide, including that of *P. vivax *[4]. Two main transporters have been studied in regard to CQR in *P. vivax*: the *P. vivax *chloroquine resistance transporter, *Pvcrt-o*, and the *P. vivax *multidrug resistance transporter, *Pvmdr1 *[5-7]. Interestingly, amino acid polymorphisms have not been associated with chloroquine resistance in *pvcrt-o *whereas *pvmdr1 *polymorphisms have been recently suggested to be associated with CQR in Southeast Asia [8]. This data indicates the involvement of other mechanisms in CQR in *P. vivax*. Likely candidates are gene amplifications and differential expression levels [9,10].

The European Network on Imported Infectious Disease Surveillance, TropNetEurop, is an electronic network of clinical sites that monitors imported infectious diseases in Europe . Since its foundation in 1999, the network has recorded 8,374 cases of malaria, of which close to 11% (930) were due to *P. vivax*. Worth mentioning, TropNetEurop covers approximately 10% of all malaria cases reported in Europe. Moreover, according to data from the Spanish National Center of Epidemiology and the Autonomous Government of Catalonia there have been 266 cases of *P. vivax *in Spain in the last six years. In addition, three cases of severe symptoms due to *P. vivax *in Europe have been reported elsewhere in the literature [11-13]. These figures illustrate the clinical-epidemiological importance of this parasitic disease in a supposedly malaria-free region.

Recently, CQR in *P. vivax *has been suggested to be associated, *albeit *not directly linked, with severe vivax malaria [14]. Here, the first clinical case of severe vivax malaria in Spain is presented. The data also indicates that clinical severity could be associated with increased expression levels of two parasite genes likely involved in chloroquine resistance, *pvmdr1 *and *pvcrt-o*.

## Methods

### Subjects

Two patients presented at Hospital Clinic in Barcelona with *P. vivax *malaria episodes. One had severe symptoms and the other mild symptoms. The patients had travelled through the Brazilian Amazon (Manaus) for 31 and 19 days, respectively, in 2007. The patient with severe symptoms was a 30-year-old Spaniard man who had previously travelled to Kenya, in 2006. Upon his return from Brazil, he presented to Hospital Clinic in Barcelona with high fever (39°C) and a Giemsa-stained thin blood film confirmed the presence of *P. vivax *at a parasitaemia of 1.8%. The patient presented acute respiratory conditions, anaemia and hyperbilirubinaemia, requiring admission to the intensive care unit. The patient with mild symptoms was a 31-year-old Spaniard man who had travelled without chemoprophylaxis and who had previously visited Mexico (2006), Vietnam (2005), and India (2004). There were no records of fever episodes from these previous trips and neither of the patients had ever been diagnosed with malaria.

Two other Brazilian patients with mild symptoms presented at the "Centro de Pesquisa em Medicina Tropical, Rondônia, Brazil in 2000. Total RNA from parasites of these patients was extracted, pooled and stored in liquid nitrogen.

### Samples

Five mL of infected red blood cells were obtained from each patient. One mL was used to purify genomic DNA following standard methodologies. The remaining blood was processed to isolate total RNA using the Trizol reagent (Invitrogen) according to the manufacturers' instructions. A recently described magnetic method for the isolation of matures stages of malaria parasites was used to concentrate and purify *P. vivax*-infected reticulocytes [15]. Giemsa-stained smears showed an absence of human leukocytes, and all the reticulocytes were infected with mature stages of the parasite (Additional file 1). Eluents were centrifuged at 800 × g for 10 minutes, supernatants discarded, and pellets used to purify total RNA. The protocol for this study was approved by the Ethical Committee of Hospital Clinic and informed consent obtained from the patients.

### Nested PCR

Nested polymerase chain reaction (PCR) was performed as previously described [16] to exclude *P. falciparum *infections. Fragments were resolved and visualized on 2% agarose gels stained with sybr green.

### qRT-PCR

Amplification reactions were performed using Power SYBR Green PCR Master Mix (Applied Biosystems) and 45 ng of template cDNA prepared from each sample. Samples were set up in duplicate and experiments were repeated independently twice. PCR products were amplified and detected on an ABI Prism 7700 (Applied Biosystems). Cycling parameters for PCR were an initial denaturation step at 95°C for 10 minutes, followed by 40 cycles of 95°C for 15 seconds, and 60°C for 1 minute. To analyse the relative transcript levels, the threshold cycle value (Ct) of each sample was used to calculate and compare the ΔCt of each sample to that of the *P. vivax *housekeeping gene Sal I β-tubulin; the ΔΔCt was also calculated as in [17] to compare the transcript levels of *pvcrt-o *and *pvmdr 1 *in the patient with severe symptoms and in the patient with mild symptoms.

## Results

### Case report

During the month of August, 2007, a 30-year-old Spanish tourist traveled through the Brazilian Amazon region of Manaus, where, due to gastric disturbances, he took an incomplete chemoprophylaxis consisting of proguanil and chloroquine. Upon his return to Spain 30 days later, he presented to a health center with an eight-day history of fever, chills and dry cough. He was diagnosed with lower respiratory tract infection and treated with amoxicillin-clavulanic acid 875/125 mg every 8 hours for 24 hours (3 doses) without resolution. On presenting a week later to Hospital Clinic in Barcelona, he had high fever (39°C) and jaundice; the tip of the spleen was palpable and chest auscultation was unremarkable. A thin blood smear of peripheral blood stained with Giemsa revealed *P. vivax *infection with a parasitaemia of 1.8%. The patient presented acute respiratory conditions requiring admission to the intensive care unit (ICU).

**Figure 1:**
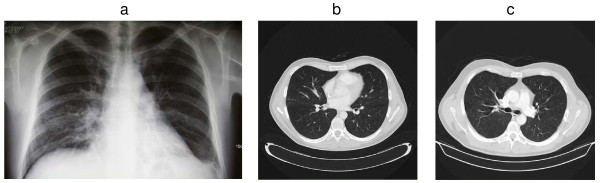
**X-ray and computed tomography (CT) scans of patient with severe *Plasmodium vivax *malaria**. Interstitial bilateral infiltrates compatible with acute lung injury in X-ray (Panel A). Infiltrates in right midzone in CT scan (panel B). Normal CT scan two months after acute episode (panel C).

In the ICU, he was haemodynamically stable and blood tests revealed pancytopaenia (haemoglobin, 10 g/L; haematocrit, 29%; platelets 25 × 10^9^/L, leukocytes, 6.7 × 10^9^/L); hyperbilirubinaemia (8.3 g/dL); γ-glutamyltransferase, 146 U/L; alkaline phosphatase, 241 U/L; and prothrombin time, 76 seconds. Renal function tests were within normal limits. The C-reactive protein level was 16 mg/dL. Arterial blood gas measurement while breathing air revealed marked hypoxia (PaO2, 63 mm Hg), normocapnia (PaCO2, 32 mm Hg), low oxygen saturation (93.7%), and a blood pH of 7.49. A chest radiograph showed bilateral interstitial infiltrates and a computed tomography (CT) scan showed right midzone alveolar shadowing without parenchymal infiltrations (Figures 1A and 1B).

**Figure 2:**
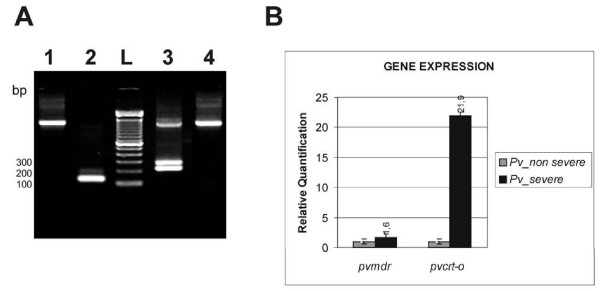
**Expression levels of chloroquine resistance genes in severe and mild *Plasmodium vivax *malaria**. In panel A, nested PCR to identify *Plasmodium *species. Amplification of gDNA from *P. falciparum *3D7 strain and *P. vivax *from severe patient, respectively, using specific *P. vivax *primers (lanes 1 and 2). Amplification of gDNA from *P. falciparum *3D7 strain and *P. vivax *from severe patient, respectively, using specific *P. falciparum *primers (lanes 3 and 4). Molecular weight ladder (lane L). A positive reaction is noted when primers for *P. falciparum *and *P. vivax *produce amplification products of 205-bp and 120-bp, respectively [16]. Molecular weight in base-pairs (bp). In panel B, relative quantification of *pvcrt-o *and *pvmdr1 *transcripts in total RNA obtained from parasites from the severe patient vs total RNA obtained from parasites from a patient from Brazil with *P. vivax *and non-severe symptoms also presenting to our hospital. The following oligonucleotide primers were designed for the real-time experiments using the Primer Express program (Applied Biosystems). Primers: F-*pvcrt-o*RT 5'-ATGTCCAAGATGTGCGACGAT-3';R-*pvcrt-o*RT 5'-CTGGTCCCTGTATGCAACTGAC-3'; F-*pvmdr1*RT 5'-AAGGATCAAAGGCAACCCA-3'; R-*pvmdr1*RT5'-TCAGGTTGTTACTGCTGTTGCTATT-3'; F-*pvtubulin*RT 5' CCAAGAATATGATGTGTGCAAGTG 3'; R-*pvtubulin*RT 5' GGCGCAGGCGGTTAGG 3'.

To exclude the possibility that the patient's severe symptoms were due to *P. falciparum*, which is sympatric with *P. vivax *in Brazil, a nested PCR using *P. falciparum*- and *P. vivax*-specific primers, was performed [16]. The results demonstrated that coinfection with *P. falciparum *could be excluded (Figure 2A). Moreover, sputum, blood, and nasopharyngeal swab samples obtained for culture were negative, as were serological tests for atypical respiratory pathogens, human immunodeficiency virus, histoplasma, coccidians, and paracoccidians. Together, these results demonstrated that the clinical symptoms were exclusively due to *P. vivax *infection.

Due to the severe condition of the patient, a five-day treatment was initiated, starting with an intravenous loading dose of quinine (1,200 mg) on the first day, followed by oral quinine (600 mg) every 8 hours plus oral doxycycline (100 mg) every 12 hours. The treatment was well-tolerated and parasitaemia became negative within three days. Platelet, erythrocyte and leukocyte levels were all within normal ranges in the following controls. Normal blood levels of glucose-6-phosphate dehydrogenase activity were detected and oral primaquine (30 mg/day) was commenced for two weeks to prevent a relapse. The patient recovered well and the chest CT findings were normal at two months (Figure 1C). There have been no recurrences during the follow-up period.

### Increased expression levels of drug resistance genes

In view of the recently suggested association of severe disease in *P. vivax *with multidrug resistance [14], expression levels of two genes suspected in having a pivotal role in mediating clinical resistance to chloroquine, *pvcrt-o *and *pvmdr1*, were determined. Significantly, the patient with severe symptoms had a 1.6-fold increase in *pvmdr1 *levels and a 21.9-fold increase in *pvcrt-o *levels compared to the other patient who presented to the same hospital with *P. vivax *infection and mild symptoms (Figure 2b). This finding was validated by analysing parasite material obtained previously from two other patients from the Brazilian Amazon who also had mild symptoms (Additional file 2).

## Discussion

There are increasing reports of severe clinical cases exclusively associated with *P. vivax *infections, involving severe anaemia, renal failure, jaundice, cerebral malaria, seizures, respiratory failure, multi-organ failure, and death [2,3]; all these complications are generally believed to be exclusively associated with severe forms of falciparum malaria. Although these same clinical severity criteria are being used for *P. vivax*, it would be desirable to conduct prospective studies to establish a precise definition of clinical severity in *P. vivax*.

The patient with severe symptoms had acute lung injury according to the definition of the American-European Consensus Conference on ARDS [18], with acute onset, bilateral changes in chest radiography, a PaO2/FiO2 ratio of ≤ 300, and absence of clinical left ventricular failure. Importantly, these respiratory complications appeared before initiation of anti-malarial drug treatment, an observation also reported in two other severe clinical cases of lung injury due to *P. vivax *[19,20]. Two main hypotheses have been proposed to explain lung damage in *P. vivax *malaria infections. The first suggests that there is no sequestration of *P. vivax*-infected reticulocytes in the deep capillaries of internal organs, postulating instead an inflammatory process due to an increase in capillary permeability associated with cytokine-induced damage in the pulmonary epithelium [21]. The second suggests cytoadherence of *P. vivax*-infected reticulocytes in lung capillaries, causing obstruction of blood flow and reduction of respiratory function before treatment and alveolar capillary damage and inflammation 24 to 48 hours after initiation of anti-malarial drug treatment [22]. The possibility that antibiotics given 24 hours before respiratory failure might have destroyed *P. vivax*-infected reticulocytes, inducing lung damage and favouring the second hypothesis, cannot formally rule out. Yet, it is clear that the lack of sequestration in *P. vivax *needs to be re-evaluated, as cytoadherence has been hypothesized to occur in both the spleen [23,24] and the lungs [22].

Chloroquine is currently the first-line treatment for *P. vivax*, but resistance has been rapidly increasing since it was first described in two cases of treatment failures in Papua New Guinea [4]. The clinical case reported here originated in Manaus, Brazil, where *P. vivax *chloroquine resistance and severe disease are now being reported [25,26]. Interestingly, after the appearance of chloroquine resistance in *P. vivax*, reports of clinical severity exclusively associated with this human malaria parasite started to appear [2]. Moreover, multidrug resistance has been recently suggested to be associated, *albeit *not directly linked, with severe disease in *P. vivax *[14]. Remarkably, higher expression levels of *pvmdr1 *and *pvcrt-o*, in particular, were found in parasites from the patient with severe clinical symptoms compared to three patients with mild symptoms. The levels were increased by up to 2.9 fold in the case of *pvmdr1 *(when including all samples) and up to 21.9 fold in the case of *pvcrt-o*. Confounding effects due to sample concentrations or contamination with *P. falciparum *or human material were excluded by using *P. vivax*-specific primers and β-tubulin as an internal control and calibrator. This data thus indicates that severe vivax disease could be associated to molecular markers.

## Conclusion

There are increasing reports of severe disease exclusively associated with vivax malaria and this study presents the first one in Spain. The use of PCR excluded incompetent microscopy, cryptic mixed infections or sequestered *P. falciparum *reinforcing the need of using PCR as a new diagnostic tool to avoid default diagnosis of severe malaria as due to *P. falciparum*. The finding on increased expression levels of parasite genes likely involved in chloroquine resistance supports to furthering explore the potential of *pvmdr1 *and particularly *pvcrt-o *as molecular markers of severe disease in *P. vivax*.

## Competing interests

The authors declare that they have no competing interests.

## Authors' contributions

All clinical aspects of these patients were studied by MP, AG, and JG.

All molecular data on DNA and RNA were produced and analysed by CFB and HdP.

PA critically reviewed the manuscript

CFB, MP, JG and HdP drafted the manuscript

## Supplementary Material

Additional file 1 ***Plasmodium vivax *purification with MACS**. Giemsa-stained thin smears representing mature forms of *Plasmodium vivax *free of human leukocytes after purification using MACS. Click here for file

Additional file 2 **Expression levels of chloroquine resistance genes in severe and mild *Plasmodium vivax *malaria**. Relative quantification of *pvcrt-o *and *pvmdr1 *transcripts in total RNA obtained from parasites of severe and non-severe *P. vivax *patients. Click here for file

## References

[B1] Price RN, Tjitra E, Guerra CA, Yeung S, White NJ, Anstey NM (2007). Vivax malaria: neglected and not benign. Am J Trop Med Hyg.

[B2] Baird JK (2007). Neglect of *Plasmodium vivax *malaria. Trends Parasitol.

[B3] Kochar DK, Das A, Kochar SK, Saxena V, Sirohi P, Garg S, Kochar A, Khatri MP, Gupta V (2009). Severe *Plasmodium vivax *malaria: a report on serial cases from Bikaner in northwestern India. Am J Trop Med Hyg.

[B4] Baird JK (2004). Chloroquine resistance in *Plasmodium vivax*. Antimicrob Agents Chemother.

[B5] Nomura T, Carlton JM, Baird JK, del Portillo HA, Fryauff DJ, Rathore D, Fidock DA, Su X, Collins WE, McCutchan TF, Wootton JC, Wellems TE (2001). Evidence for different mechanisms of chloroquine resistance in 2 Plasmodium species that cause human malaria. J Infect Dis.

[B6] Brega S, Meslin B, de Monbrison F, Severini C, Gradoni L, Udomsangpetch R, Sutanto I, Peyron F, Picot S (2005). Identification of the *Plasmodium vivax *mdr-like gene (pvmdr1) and analysis of single-nucleotide polymorphisms among isolates from different areas of endemicity. J Infect Dis.

[B7] Sa JM, Nomura T, Neves J, Baird JK, Wellems TE, del Portillo HA (2005). *Plasmodium vivax*: allele variants of the mdr1 gene do not associate with chloroquine resistance among isolates from Brazil, Papua, and monkey-adapted strains. Exp Parasitol.

[B8] Suwanarusk R, Russell B, Chavchich M, Chalfein F, Kenangalem E, Kosaisavee V, Prasetyorini B, Piera KA, Barends M, Brockman A, Lek Uthai U, Anstey NM, Tjitra E, Nosten F, Chen Q, Price RN (2007). Chloroquine resistant *Plasmodium vivax*: in vitro characterisation and association with molecular polymorphisms. PLoS ONE.

[B9] Imwong M, Pukrittayakamee S, Pongtavornpinyo W, Nakeesathit S, Nair S, Newton P, Nosten F, Anderson TJ, Dondorp A, Day NP, White NJ (2008). Gene amplification of *Plasmodium vivax *multidrug resistance 1 gene in Thailand, Laos, and Myanmar. Antimicrob Agents Chemother.

[B10] Sa JM, Yamamoto MM, Fernandez-Becerra C, de Azevedo MF, Papakrivos J, Naude B, Wellems TE, Del Portillo HA (2006). Expression and function of pvcrt-o, a Plasmodium vivax ortholog of pfcrt, in *Plasmodium falciparum *and Dictyostelium discoideum. Mol Biochem Parasitol.

[B11] Kumar S, Melzer M, Dodds P, Watson J, Ord R (2007). *P. vivax *malaria complicated by shock and ARDS. Scand J Infect Dis.

[B12] Lawn SD, Krishna S, Jarvis JN, Joet T, Macallan DC (2003). Case reports: pernicious complications of benign tertian malaria. Trans R Soc Trop Med Hy.

[B13] Tanios MA, Kogelman L, McGovern B, Hassoun PM (2001). Acute respiratory distress syndrome complicating *Plasmodium vivax *malaria. Crit Care Med.

[B14] Tjitra E, Anstey NM, Sugiarto P, Warikar N, Kenangalem E, Karyana M, Lampah DA, Price RN (2008). Multidrug-resistant *Plasmodium vivax *associated with severe and fatal malaria: a prospective study in Papua, Indonesia. PLoS Medicine.

[B15] Trang DT, Huy NT, Kariu T, Tajima K, Kamei K (2004). One-step concentration of malarial parasite-infected red blood cells and removal of contaminating white blood cells. Malar J.

[B16] Snounou G, Singh B (2002). Nested PCR analysis of Plasmodium parasites. Methods Mol Med.

[B17] Livak KJ, Schmittgen TD (2001). Analysis of relative gene expression data using real-time quantitative PCR and the 2(-Delta Delta C(T)) Method. Methods.

[B18] Bernard GR, Artigas A, Brigham KL, Carlet J, Falke K, Hudson L, Lamy M, Legall JR, Morris A, Spragg R (1994). The American-European Consensus Conference on ARDS. Definitions, mechanisms, relevant outcomes, and clinical trial coordination. Am J Resp Crit Care Med.

[B19] Lomar AV, Vidal JE, Lomar FP, Barbas CV, de Matos GJ, Boulos M (2005). Acute respiratory distress syndrome due to vivax malaria: case report and literature review. Braz J Infect Dis.

[B20] Munteis E, Mellibovsky L, Marquez MA, Minguez S, Vazquez E, Diez A (1997). Pulmonary involvement in a case of *Plasmodium vivax *malaria. Chest.

[B21] Price L, Planche T, Rayner C, Krishna S (2007). Acute respiratory distress syndrome in *Plasmodium vivax *malaria: case report and review of the literature. Trans R Soc Trop Med Hyg.

[B22] Anstey NM, Handojo T, Pain MC, Kenangalem E, Tjitra E, Price RN, Maguire GP (2007). Lung injury in vivax malaria: pathophysiological evidence for pulmonary vascular sequestration and posttreatment alveolar-capillary inflammation. J Infect Dis.

[B23] Fernandez-Becerra C, Yamamoto MM, Vencio RZ, Lacerda M, Rosanas-Urgell A, Del Portillo HA (2009). *Plasmodium vivax *and the importance of the subtelomeric multigene vir superfamily. Trends Parasitol.

[B24] del Portillo HA, Lanzer M, Rodriguez-Malaga S, Zavala F, Fernandez-Becerra C (2004). Variant genes and the spleen in *Plasmodium vivax *malaria. Int J Parasit.

[B25] de Santana Filho FS, Arcanjo AR, Chehuan YM, Costa MR, Martinez-Espinosa FE, Vieira JL, Barbosa MG, Alecrim WD, Alecrim MG (2007). Chloroquine-resistant *Plasmodium vivax*, Brazilian Amazon. Emerging Infect Dis.

[B26] Lacerda MV, Alexandre MA, Santos PD, Arcanjo AR, Alecrim WD, Alecrim MG (2004). Idiopathic thrombocytopenic purpura due to vivax malaria in the Brazilian Amazon. Acta Trop.

