# Supplementary material for: Increased expression levels of the pvcrt-o and pvmdr1 genes in a patient with severe Plasmodium vivax malaria
Source: Malar J. 2009 Apr 2;8:55. doi: 10.1186/1475-2875-8-55 (PMC2682795; doi:10.1186/1475-2875-8-55)
Supplement: Additional file 1 — Plasmodium vivax purification with MACS. Giemsa-stained thin smears representing mature forms of Plasmodium vivax free of human leukocytes after purification using MACS. [file 1475-2875-8-55-S1.pdf]

Additional file 1

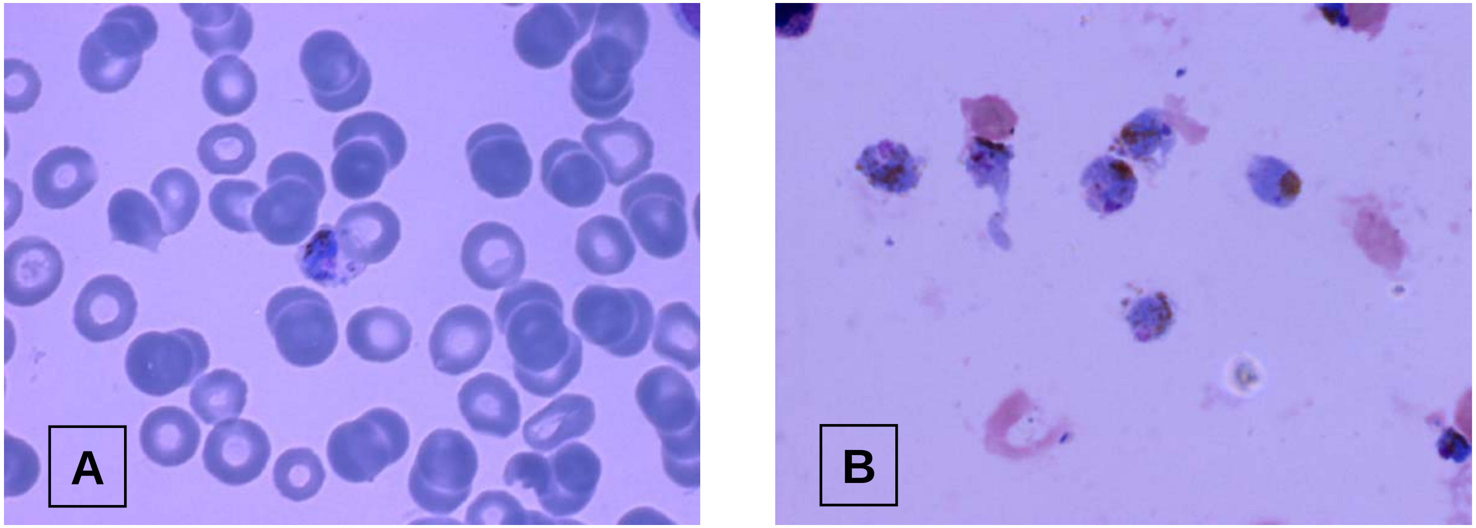

***Plasmodium vivax* purification with MACS (magnification, ×100).** Giemsa-stained thin smears representing mature forms of *Plasmodium vivax* from the severe patient before (A) and after (B) purification. Briefly, prior to purification, the MACS columns (25 LD columns; Miltenyi Biotec) were filled with incomplete RPMI. Blood from patients was washed and resuspended in RPMI and applied to columns assembled in a Quadro MACS magnetic support (Miltenyi Biotec). Culture medium was then added until the eluent from the columns became almost colorless. Columns were removed from the magnetic support and the cells retained in the column were eluted with 1 mL of culture medium.
