# Supplementary material for: Increased expression levels of the pvcrt-o and pvmdr1 genes in a patient with severe Plasmodium vivax malaria
Source: Malar J. 2009 Apr 2;8:55. doi: 10.1186/1475-2875-8-55 (PMC2682795; doi:10.1186/1475-2875-8-55)
Supplement: Additional file 2 — Expression levels of chloroquine resistance genes in severe and mild Plasmodium vivax malaria. Relative quantification of pvcrt-o and pvmdr1 transcripts in total RNA obtained from parasites of severe and non-severe P. vivax patients. [file 1475-2875-8-55-S2.pdf]

Additional file 2

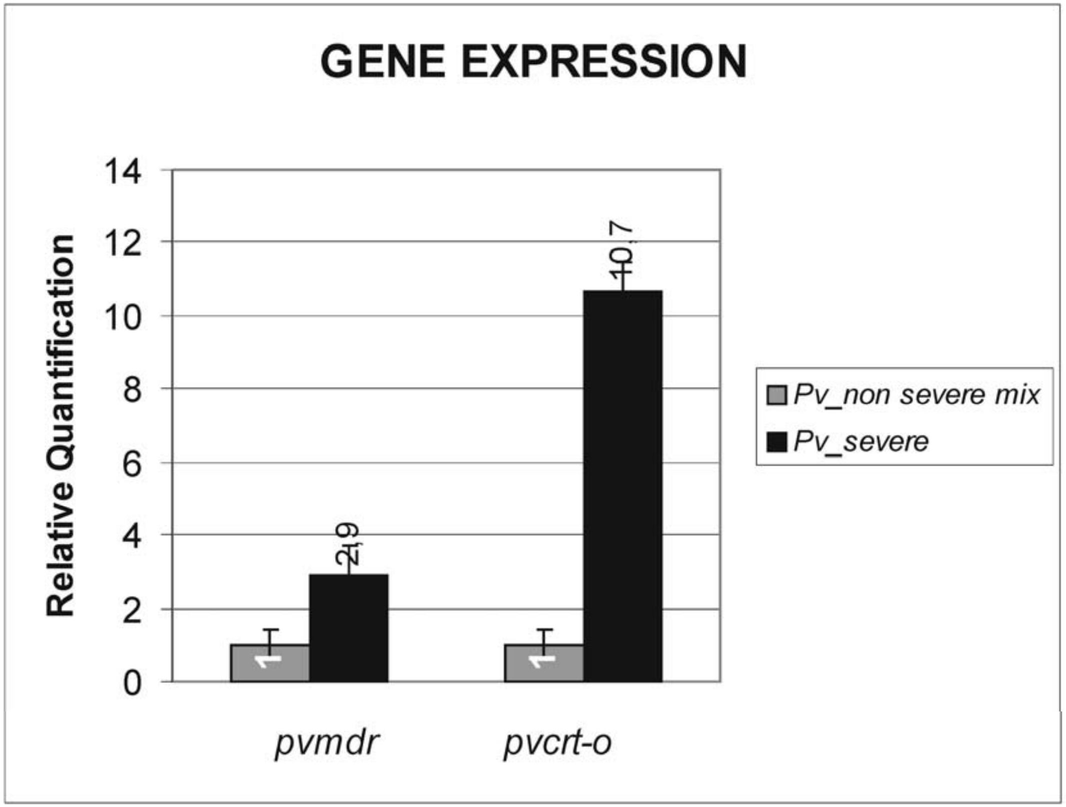

Expression levels of chloroquine resistance genes in severe and mild P*lasmodium vivax* malaria. Relative quantification of *pvcrt-o* and *pvmdr1* transcripts in total RNA obtained from parasites from the severe patient vs a mix of total RNA obtained from parasites from two patients from Brazil with *P. vivax* and non-severe symptoms. Primers and conditions are described in the Methods section and in Figure 2.
